# Adverse childhood experiences, re-trafficking vulnerability, and mental health outcomes among women survivors of human trafficking in Kampala, Uganda: A cross-sectional analysis

**DOI:** 10.1371/journal.pone.0334419

**Published:** 2025-11-18

**Authors:** Violet Nkwanzi, Robert M. Bulamba, David Okech, Catherine Carlson, Emmanuel Kyasanku, Sylvia Namakula, Deneen Evans, Alex Daama, Stephen Mugamba, Dorah Akello, Emmanuel Menya, Jackline B. Nammanda, James Nkale, Rogers Kasirye, Fred Nalugoda, Godfrey Kigozi, Gertrude Nakigozi

**Affiliations:** 1 Department of Social Work, College of Health Sciences, East Tennessee State University (ETSU), United States of America; 2 Department of Epidemiology and Behavioral Research, Africa Medical and Behavioral Sciences Organization (AMBSO), Kampala, Uganda,; 3 School of Social Work, University of Georgia, United States of America; 4 Department of Social Work, School of Social Work, University of Alabama, United States of America; 5 Department of Research, Centre on Anti-trafficking Research and Empowerment (CATRE), Kampala, Uganda,; 6 Department of Research, Healing and Resilience after Trauma (HART), Kampala, Uganda,; 7 Department of Research, Uganda Youth Development Link (UYDEL), Kampala, Uganda; Taipei Veterans General Hospital, TAIWAN

## Abstract

**Background:**

Globally, human trafficking disproportionately affects women and girls, exposing them to severe exploitation and long-term psychological, social, and economic harm. While global efforts to prevent trafficking have intensified, the risk of re-trafficking remains a critical yet understudied issue in low-resource settings, particularly in Uganda. Uganda’s limited data on the effects of adverse childhood and re-trafficking vulnerabilities on the long-term mental health outcomes of survivors of trafficking limits the design of evidence-based interventions to improve survivors’ health. Our study examined the link between Adverse Childhood Experiences (ACE), re-trafficking vulnerability, and lasting mental health consequences among female survivors of human trafficking in Uganda

**Methods:**

A cross-sectional study was conducted among 350 female survivors of human trafficking in Kampala, Central Uganda, in January 2025. Trained female research assistants conducted one-on-one interviews in English or Luganda, a local language. Data on participants’ socio-demographic characteristics, adverse childhood experiences (ACE-IQ), human trafficking vulnerability (AHTST), anxiety (GAD-7), depression (PHQ-9), and PTSD were collected. Bivariate and multivariable modified regression models with robust standard errors were performed using Stata version 17.0 for analysis

**Results:**

Of the 350 female survivors of trafficking interviewed in Central Uganda, more than half (63.7%) reported experience of ACEs, 63.4% screened positive for human trafficking vulnerability, 57.1%, 56%, and 40.9% identified with anxiety, depression, and PTSD symptoms, respectively. Experience of ACEs strongly correlated with a 5%, 4%, and 6% increased risk of experiencing anxiety (aRR = 1.05, 95% CI: 1.02-1.08), depression (aRR = 1.04, 95% CI: 1.01-1.06), and PTSD (aRR =1.06, 95% CI: 1.03-1.09) symptoms, respectively. Additionally, age and education were strongly linked to a heightened risk of experiencing mental health (MH) symptoms among this population. Furthermore, trafficking vulnerabilities were paradoxically associated with lower symptom severity of all three MH conditions in this population

**Conclusion:**

This study highlights the strong link between adverse childhood experiences (ACEs), re-trafficking vulnerability, and MH risks among trafficking survivors in Uganda. High ACE exposure significantly increases anxiety, depression, and PTSD risk. The results reveal the immediate need for trauma-informed interventions addressing ACEs and mental health to reduce re-trafficking risks and promote survivor resilience. Unexpectedly, greater trafficking vulnerability correlated with lower symptom severity, warranting further investigation.

## Introduction

Human trafficking affects several women and girls around the world, subjecting them to exploitation in various forms, including sex trafficking and forced labor. The consequences of trafficking extend beyond immediate physical harm to long-term psychological, social, and economic impacts [1]. While global efforts to prevent trafficking have intensified, the risk of re-trafficking remains a critical yet underexplored issue, particularly in low-resource settings such as Uganda. Survivors of trafficking, especially those with adverse childhood experiences (ACEs), face heightened vulnerability to re-trafficking due to compounded socio-economic hardships, psychological distress, and systemic gaps in protective structures [2–5].

Uganda is both a source and transit country for human trafficking, with significant numbers of women and girls falling victim to exploitation domestically and internationally [6]. The primary drivers of trafficking in Uganda include poverty, gender-based violence (GBV), unemployment, and internal displacement due to conflict and natural disasters [7]. Women and girls in urban centers such as Kampala face increased risks of exploitation, often under the guise of employment opportunities in domestic work, hospitality, and commercial sex work [8]. The 2022 Uganda Annual Trafficking in Persons Report indicated that 1,200 trafficking cases were identified, with women and girls comprising the majority of victims [9]. Government and non-governmental organizations provide rehabilitation and reintegration services to survivors. However, many survivors remain at risk of re-trafficking due to limited economic opportunities, stigmatization, and unresolved psychological trauma [10].

Additionally, the risk of trafficking and other forms of exploitation is compounded by experiences of adverse childhood adversities(ACEs) [11]. ACEs encompass childhood exposure to domestic violence, abuse, neglect, substance use in the household, and parental separation [11–13].In Uganda, studies indicate that a significant proportion of children experience ACEs, with girls disproportionately affected due to gendered vulnerabilities [14].

For female trafficking survivors, past ACEs contribute to an increased likelihood of re-trafficking by undermining resilience and perpetuating cycles of victimization [15]. Childhood adversity disrupts emotional regulation and decision-making, making individuals more susceptible to coercion and exploitation [10]. Survivors facing economic instability and social marginalization, which are key aspects of re-trafficking vulnerability, may experience heightened anxiety, depression, and PTSD due to chronic stress and uncertainty [2–5]. Understanding these interactions is crucial for designing interventions that address both trauma and economic precarity. In Uganda, where social support structures for survivors are often weak, women with extensive ACEs may struggle to rebuild their lives, further predisposing them to re-exploitation [10].

The psychological impact of human trafficking is profound, with survivors experiencing increased rates of depression, post-traumatic stress disorder (PTSD), and anxiety [8]. Studies have shown that trafficking-related trauma, compounded by pre-existing childhood adversity, results in persistent mental health challenges that hinder reintegration and self-sufficiency [4]. In Uganda, where access to mental health care is limited, trafficking survivors often lack the necessary psychological support to recover from their experiences [16].

By exploring ACEs, re-trafficking vulnerability, and mental health outcomes among female survivors of human trafficking in Kampala, Uganda, this study aims to provide actionable insights for policymakers, service providers, and communities, supporting the development of targeted reintegration programs that enhance survivors’ psychological well-being, economic stability, and resilience against re-exploitation.

## Materials and methods

### Study design, study population, and setting

Data for this cross-sectional research study was collected between 16^th^ – 30^th^ January 2025 from 350 consenting female survivors of human trafficking at 7 sites operated by Uganda Youth Development Link (UYDEL) −5 sites and Set Her Free (SHF) −2 sites, agencies serving survivors of human trafficking in Kampala, Central Uganda. Convenience sampling was employed to select 350 eligible women survivors of sex and labor trafficking who were actively receiving rehabilitation services at these sites. Eligible participants were women aged 18–35 who provided written informed consent. Survivors with physical or cognitive impairments that prevented basic functioning, as assessed by partner agency staff, were excluded from the study.

### Procedures

The current study received ethical approval from East Tennessee State University (C0724.10S), Clarke International University (CLARKE-2024–1141), and the registered with the Uganda National Council for Science and Technology (SS3451ES). Caseworkers at Uganda Youth Development Link (UYDEL) and SET HER FREE (SHF) conducted screening of participants based on agency records. The research team then verified eligibility, ensuring that participants were survivors of sex or labor trafficking. Female research assistants (RAs) trained by the Africa Medical and Behavioral Sciences Organization (AMBSO) conducted face-to-face interviewer-administered quantitative interviews. The RAs were fluent in English and Luganda – the local language and interviews were conducted in the participant’s preferred language. Each interview lasted approximately 60 minutes and took place in a secure location at UYDEL or SHF to maintain confidentiality. The informed consent documents were translated to the local language, all eligible participants provided informed consent and were given a copy of the consent form for their personal use. Participants were reimbursed according to the approved study protocol.

### Measures and data collection

Socio-demographic characteristics: Participant’s data such as their age, marital status, education level, religion, and area of residence was collected.

### Adverse Childhood Experiences International Questionnaire (ACE-IQ)

We adapted the 10-item Adverse Childhood Experiences-International Questionnaire (ACE-IQ) to examine Adverse childhood experiences (ACEs) [17] in this population. The Items comprised of emotional, physical, and/or sexual abuse; emotional and/or physical neglect; bullying; having a house member who was incarcerated; living with a household member who has had a substance use problem or a mental illness; experiencing the death of a parent; witnessing domestic violence; divorce, or separation; witnessing interpersonal violence in the community; and exposure to collective violence such as war, terrorism, organized violence, or political conflict. Each item was coded as a binary variable, with a score of 1 indicating exposure to the experience and 0 indicating no exposure. Responses from the 10 binary questions were summed up to calculate the cumulative number of ACEs, with scores ranging from 0–10. The Cronbach’s alpha obtained was 0.53, closely consistent with the reliability and internal consistency of 0.67 reported in previous work in Sub-Saharan Africa (SSA) [18]. The average participant’s score on all the 10 items was 8.0, and this was used as the cut-off score for identifying the participants who screened positive for ACEs, as previously used in other work as a screening criteria for patient referral for clinical assessment and care [19–21].

### Trafficking vulnerability (risk of re-trafficking)

Human trafficking vulnerability was measured using the 8-item Adult Human Trafficking Screening Tool (AHTST) [22], with responses coded as yes/no with any experience of the event categorized as “1” and no experience of the event categorized as “0”. This assessed the survivor’s risk of re-trafficking. The items used include; using lies to be tricked into accepting a job that doesn’t exist, getting trapped in a job or situation they never wanted, repaying a person who provided them with transportation, a place to stay, money, doing unfair, unsafe, or even dangerous work or remain in hazardous situation being denied their own identification or travel documents, working for someone or spend time with someone who does not let them contact their family, spend time with their friends, or go where they want, living where they work and they’re not allowed to live elsewhere, being told to lie about their situation, including the kind of work they do, hurt or threatened, or threats are made to their family or loved ones, or they are forced to do things they do not want to do to make money for someone else or to pay off a debt to them. Participants who answered YES to any items indicated their current or future risk of re-trafficking. Upon their consent, these were referred to trained authorities for psychosocial support.. The individual responses were summed up to calculate the cumulative number of re-trafficking vulnerabilities (composite), with the scale ranging from 0–8, and a Cronbach’s alpha of 0.71 was obtained. The average human trafficking vulnerability score was 5.0. We used the scale’s average score to determine participants who screened positive for re-trafficking vulnerabilities, and this approach has been previously used in other fields as a scoring mechanism for establishing cut-off points to support referral of patients for further clinical assessment and care [20,21,23]. Although the AHTST has not yet been validated, the test reliability and internal consistency of the tool’s items signal its ability to reliably identify adults who may have been trafficked or at risk of being trafficked in this setting.

### Anxiety (GAD-7)

The 7-items Generalized Anxiety Disorder scale (GAD-7) tool was used to measure symptoms of anxiety on Likert scale. The GAD-7 is a validated screening tool [8] and has been previously used to assess the experience of anxiety symptoms in resource-limited settings. The 7-items evaluated the severity of symptoms with responses; 0- Not at all to 3- Nearly every day, with a maximum possible range of 0–21 [24]. Experience of anxiety symptoms was assess using questions such as; “feeling nervous, anxious or on edge” and “not being able to stop or control worrying” With a recall period of 30 days. The scale produced a reliability and internal consistency Cronbach’s alpha = 0.81, consistent with ones obtained in previous studies [24,25]. Forms of anxiety were classified as no anxiety (GAD-7 score <10) or generalized anxiety disorder (GAD-7 score ≥10). Consistent with the findings of other studies [19], we used a cut-off score of 8, which is generally accepted as a positive screen for anxiety symptoms.

### Depression (PHQ-9)

We used the Patient Health Questionnaire (PHQ-9), a 9-item validated measure [26,27] used before in Uganda [28,29] among adult populations, in SSA countries among people living with HIV [18], and in high-income countries among adults and survivors of trauma.[30,31]. Depressive symptoms were measured on a 3-point Likert-type scale ranging from 0-Not at all to 3-Nearly every day, with the total score reflecting a severity score for each participant. In our study, the PHQ-9 scale showed strong internal consistency, with a (Cronbach’s alpha = 0.84), consistent with findings from earlier research, which suggests strong internal consistency of the measure’s items in our sample. Total scores were categorized as no depression (0–5), mild to moderate depression (5–14), or severe depression (>14), and we used a cut-off of 10 scores for positive screen, consistent with other studies where the PHQ-9 has been applied in similar populations [19].

### Post-Traumatic Stress Disorder (PTSD-Scale)

We used the posttraumatic stress disorder, a Diagnostic and Statistical Manual of Mental Health (MH) DSM-PCL-5 scale, for measuring PTSD symptoms among survivors of trauma. PTSD-20 is a standard validated screening tool consisting of 20 items [32] that assessed PTSD symptoms on the following 4 domains: the original clusters of intrusion, avoidance, and hyper-arousal and the added cluster of negative alterations in cognition and mood with three items (blame, negative emotions, and reckless or self-destructive behavior) [32]. Item scores were summed for an overall severity score for individual symptom cluster sums. Items are rated on a four-point Likert scale (1 = not at all, 4 = very often), and the items in the scale were summed up to obtain the minimum (0) and maximum (80) possible score [33]. In this study, the measure presented a good internal consistency (Cronbach’s alpha = 0.92), which consistently aligns with the internal consistency of 0.91, 0.94 obtained in studies in SSA [32,34], and 0.79 and 0.95 in studies elsewhere [35,36]. The DSM-PCL-5 validation studies suggest a cut-off score of 33 for a diagnosis of PTSD [34,37]. Similarly, in our study, we used a cut-off score of 33 for PTSD diagnosis.

### Statistical analysis

We performed analyses using Stata version 17.0. Descriptive analyses were performed to describe our study sample characteristics, and factors associated with ACEs, human trafficking vulnerability, and mental health (MH) symptoms (depression, anxiety, and PTSD). For continuous variables, we interpreted their mean and standard deviation (SD). For categorical variables, we interpreted frequencies and percentages. The prevalence of anxiety, depression, and PTSD symptoms was determined as the number of people who screened positive for each divided by the total number of people in the study sample. A similar approach was used to obtain the prevalence ACEs and human trafficking vulnerability. Variables with a *p*-value < 0.1 in the univariate analysis were included in the multivariable models. For each outcome, we fitted four different models, using the modified Poisson regression model [38] and linear regression models [38] with robust standard errors to adjust for non-constant variance of the error terms to produce more reliable statistically significant tests [38] to estimate the relative risk (RR) of the outcomes associated with the covariates. Alternately, we specified ACEs in model 1, or re-trafficking vulnerability in model2 as the primary explanatory variables of interest. Each outcome variable was scored with 1 representing the presence of anxiety, depression, and/or PTSD and zero (0) representing the absence of symptoms. The similar approach was applied to ACEs and trafficking vulnerabilities, and variables such as age, marital status, education level, place of residence, and religion were included in the model [18,39]. The two primary explanatory variables, (ACEs and re-trafficking vulnerabilities) were included in separate regression models to control for multicollinearity between ACEs and trafficking vulnerabilities. For sensitivity, we used the linear regression models to estimate the association between ACEs and mental health outcomes in model3, and then between trafficking vulnerabilities and mental health outcomes in Model4, specifying depression, anxiety, and PTSD symptoms severity as continuous variables but rather not as binary outcome variables. This additional analysis was conducted because mental health is generally and contextually measured on a continuum and not as a binary scale [18].

## Results

### Characteristics of the study participants

Out of the 350 participants surveyed, more than half (70.3%) were aged between 18 and 24 years, with a mean age of 23 years (SD = 4.1). Most (80%) were single, while 13.1% were married and 6.9% divorced. Nearly half (48%) of the participants had attained only a primary level of education, while 28.6% had no formal education, and 23.4% had at least a secondary-level education. Regarding living arrangements, 78% lived with their families or in their own homes, 19.7% stayed with a friend or at their workplace, and 2.3% resided in a shelter. Majority (33.1%) of the participants were Catholics, with 28.9% and 20.6%, being Moslems and Pentecostal/saved respectively. Participants exhibited high levels of mental health concerns, with mean anxiety (M = 9.86, SD = 4.41), depression (M = 10.35, SD = 4.73), and post-traumatic stress disorder (PTSD) (M = 30.31, SD = 14.71) symptom scores. Exposure to adverse childhood experiences (ACEs) was prevalent, and an average of 8.07 ACEs (SD = 2.33) was reported in this population. Trafficking vulnerability was also notably high, with an average score of 5.0 (SD = 2.2) (Table 1).

**Table 1:** Characteristics of the study participants.

Variable	N (%) or Mean (SD)	
**Age in years**		23 (4.07)
18-24 years	246 (70.3%)	
25-35 years	104 (29.7%)	
**Marital Status**		
Married	46 (13.1%)	
Single	280 (80%)	
Divorced	24 (6.9%)	
**Education Level**		
No education	100 (28.6%)	
Primary education	168 (48%)	
Secondary education and above	82 (23.4%)	
**Place of stay/sleeping area**		
Shelter	8 (2.3%)	
With a family member or own home	273 (78.0%)	
With a friend or workplace	69 (19.7%)	
**Religion**		
Catholics	116 (33.1%)	
Moslems	101 (28.9%)	
Pentecostal/Saved	72 (20.6%)	
Others (Anglican)	61 (17.4%)	
**Mental health symptoms and Trauma events**		
Anxiety (GAD-7)		8.86 (4.41)
Depression (PHQ-9)		10.35 (4.73)
Post-traumatic Stress Disorder (PTSD)		30.31 (14.71)
Adverse Childhood vulnerabilities (ACE-IQ)		8.07 (2.33)
Human Trafficking Vulnerabilities (AHTST)		5.0 (2.2)

### Prevalence of anxiety, depression, PTSD, ACEs and trafficking vulnerability

Overall, 57.1% (200/350) of participants screened positive for elevated anxiety symptoms (Table 2), with higher (68.5%) rates observed among participants aged 18–24 years compared to those aged 25–35 years (31.5%) (X² = 0.71, p = 0.399). Similarly, 56.0% (n = 196) of participants screened positive for depression, with younger participants reporting significantly higher (62.8%) experience of depression compared to older ones (37.2%) (X² = 12.09, p = 0.001). PTSD symptoms were present in 40.9% (n = 143) of participants, with a greater prevalence among those aged 18–24 years (67.1%) compared to those aged 25–35 years (32.9%). Exposure to ACEs was reported by 63.7% (n = 223) of participants, with the majority being younger women (71.8% vs. 28.3%). Additionally, 63.4% (n = 222) of participants screened positive for trafficking vulnerability, with a significantly higher proportion of younger participants (65.4%) compared to older participants (34.6%) (X² = 4.60, p = 0.032), indicating a higher risk of re-trafficking among younger survivors of trafficking (Table 2).

**Table 2:** Prevalence of Anxiety, Depression, PTSD, ACEs, and Human Trafficking Vulnerabilities, stratified by age (N = 350).

Variable	Overall	18-24 years	25-35 years	X^2^	P-value
	n (%)	n (%)	n (%)		
**Anxiety**				0.71	0.399
Positive screen	200 (57.1)	137 (68.5)	63 (31.5)		
Mean (SD)	8.9(4.4)	8.5(4.1)	9.7(5.0)		
**Depression**				12.09**	0.001
Positive screen	196(56.0)	123(62.8)	73(37.2)		
Mean (SD)	10.4(4.7)	9.7(4.4)	11.9(5.1)		
**PTSD**				1.15	0.283
Positive screen	143 (40.9)	96(67.1)	47(32.9)		
Mean (SD)	30.3(14.7)	30.2(14.7)	30.6(14.8)		
**ACEs**				0.63	0.427
Positive screen	223 (63.7)	160(71.8)	63(28.3)		
Mean (SD)	8.1(2.3)	8.1(2.3)	8.0(2.5)		
**Human Trafficking Vulnerabilities**				4.60**	0.032
Positive screen	222 (63.4)	123(65.4)	65(34.8)		
Mean (SD)	5.0(2.2)	4.4(2.2)	5(2.1)		

Depression was measured using the PHQ-9, with a score ≥10 = positive screen for symptoms of depression. Anxiety was measured using the GAD-7 scale, with a score ≥8 = positive screen for symptoms of anxiety. PTSD was measured using the DSM-PCL-5 scale, with a score ≥33 = indicative of severe stress. ACEs were measured using the 10-item ACE-IQ, with a score ≥8 indicative ofpositive screen for ACEs. The risk of re-trafficking was measured using the IAHTST; a score ≥5.0  = a positive screen for current/future re-trafficking vulnerability. **P < 0.01.

### ACEs, trafficking vulnerability, and anxiety symptoms

Table 3 presents the results of the modified Poisson regression models with robust standard errors and linear regression models. In Model 1, each ACE was associated with a 5% increased risk of experiencing anxiety symptoms (aRR = 1.05; 95% CI: 1.02–1.08; p < 0.001). Age was also significantly associated with anxiety symptoms, with a 2% increased risk associated with each additional year (aRR = 1.02; 95% CI: 1.00–1.03; p = 0.011). Having at least a secondary education and experiencing trafficking increased the risk of anxiety symptoms by 20% (aRR = 1.20; 95% CI: 1.05–1.39; p = 0.009). Anxiety symptom severity as a continuous outcome yielded similar estimates. In Model 3, anxiety severity was significantly associated with ACEs (b = 0.44 per ACE, 95% CI = 0.22–0.66, p = 0.001), age (b = 0.15, 95% CI: 0.03–0.27, p = 0.015), and secondary education and above (b = 1.67, 95% CI: 0.39–2.95, p = 0.011). Model 4 indicated that trafficking vulnerability was negatively associated with anxiety symptom severity (b = −0.63 per unit vulnerability, 95% CI: −0.84 to −0.43, p < 0.001) (Table 3).

**Table 3:** Correlates of moderate to severe anxiety Symptoms (N = 350).

Variable	Adjusted relative risk ratio (95% confidence interval (CI), p-value		Unstandardized regression coefficient (95% CI), p-value	
	Model 1	Model 2	Model 3	Model 4
ACEs per unit	1.05 (1.02, 1.08), < 0.001***		0.44 (0.22, 0.66), < 0.001***	
Trafficking Vulnerabilities/Unit		0.93 (0.91, 0.95), < 0.001***		−0.63 (−0.84, −0.42), < 0.001***
**Marital Status**				
**Single**	1.00	1.00	1.00	1.00
Married	0.95(0.80, 1.12),0.531	0.97(0.83, 1.15),0.758	−0.45(−1.84, 0.94),0.522	−0.23(−1.57(1.12),0.741
Widowed	0.93(0.76, 1.13),0.443	0.92(0.76, 1.11),0.371	−0.71(−2.53, 1.10),0.441	−0.75(−2.49, 0.99),0.398
**Religion**				
Christians	1.00	1.00	1.00	1.00
Moslems	1.07(0.94, 1.22),0.282	1.11(0.97, 1.26),0.122	0.66(−0.49, 1.81),0.261	0.87(−0.26(2.00),0.132
Pent/ Saved	0.93(0.80, 1.08), 0.338	0.96(0.83, 1.11),0.575	−0.53(−1.75, 0.68),0.389	−0.33(−1.53, 0.87),0.594
Others	1.12(0.98, 1.29),0.101	1.11(0.97, 1.27),0.132	1.05(−0.22, 2.32),0.106	0.89(−0.34, 2.12),0.155
**Age**, Per year	1.02(1.00, 1.03),0.011*	1.01(0.99, 1.02),0.160	0.15(0.03, 0.27),0.015*	0.08(−0.04, 0.20),0.177
**Place of residence**				
In Shelter	1.00	1.00	1.00	1.00
With a family member/own home	0.89(0.67, 1.19),0.433	0.97(0.71, 1.31),0.830	−1.27(−4.29, 1.75),0.409	−0.47(−3.69, 2.74),0.773
With a friend/workplace	0.99(0.74, 1.34),0.961	1.02(0.74, 1.41),0.888	−0.33(−3.46, 2.81),0.838	0.02(−3.29, 3.34),0.991
**Education Level**				
No education	1.00	1.00	1.00	1.00
Primary education	1.02(0.90, 1.15),0.791	0.99(0.88, 1.12),0.925	0.14(−0.88, 1.16),0.793	−0.01(−1.00, 0.99),0.989
Sec education and above	1.20 (1.05, 1.39), 0.009**	1.19(1.03, 1.36),0.014*	1.67(0.39, 2.95),0.011*	1.55(0.29, 2.81),0.015*

*Others = Anglicans; *p < 0.05; **p < 0.01; ***p < 0.001.

### ACEs, trafficking vulnerability, and depression symptoms

The analyses in Table 4 show that ACEs, trafficking vulnerabilities, age, and higher education levels were associated with severe forms of depression. In Model 1, each additional ACE was associated with a 4% increased risk of severe depression (aRR = 1.04, 95% CI: 1.01–1.06, p = 0.002). Each additional year of age was associated with a 3% increased risk (aRR = 1.03, 95% CI: 1.02–1.04, p < 0.001), and having a secondary education or higher was associated with a 9% increased risk of severe depression (aRR = 1.09, 95% CI: 0.96–1.24, p < 0.001). However, unlike ACEs, in Model 2, each additional point on the trafficking vulnerability scale was associated with a 6% reduced risk of severe depression (aRR = 0.94, 95% CI: 0.92–0.96, p < 0.001). In Models 3 and 4, ACEs, age, and trafficking vulnerabilities remained significantly associated with depression symptoms: ACEs (b = 0.40 per unit, 95% CI: 0.15–0.65, p = 0.002), age (b = 0.29 per year, 95% CI: 0.16–0.41, p < 0.001), and trafficking vulnerabilities (b = −0.63, 95% CI: −0.85 to −0.42, p < 0.001) (Table 4).

**Table 4:** Correlates of moderate to severe depression Symptoms (N = 350).

Variable	Adjusted relative risk ratio (95% confidence interval (CI), p-value		Unstandardized regression coefficient (95% CI), p-value	
	Model 1	Model 2	Model 3	Model 4
ACEs per unit	1.04 (1.01, 1.06), 0.002**		0.40 (0.15, 0.65), 0.002**	
Trafficking Vulnerabilities/Unit		0.94 (0.92, 0.96), < 0.001***		−0.63 (−0.85, −0.42), < 0.001***
**Marital Status**				
Single	1.00	1.00	1.00	1.00
Married	0.95(0.83, 1.09),0.512	0.98(0.85, 1.13),0.748	−0.51(−1.91, 0.88),0.470	−0.29(−1.69, 1.11),0.688
Widowed	0.90(0.83, 1.09),0.512	0.89(0.76, 1.06),0.191	−1.09(−2.94, 0.74),0.241	−1.16(−2.89, 0.58),0.190
**Religion**				
Christians	1.00	1.00	1.00	1.00
Moslems	1.04(0.92, 1.17),0.529	1.06(0.95, 1.19),0.295	0.41(−0.79, 1.61),0.506	0.60(−0.56, 1.76),0.311
Pent/ Saved	0.94(0.82, 1.06),0.306	0.96(0.84, 1.09),0.499	−0.61(−1.84, 0.63),0.338	−0.41(−1.65, 0.83),0.513
Others	1.1(0.98, 1.26),0.111	1.09(0.97, 1.25),0.156	1.23(−0.30, 2.58),0.122	0.96(−0.44, 2.37),0.180
**Age**, Per year	1.03 (1.02, 1.04), < 0.001***	1.02 (1.01, 1.03), < 0.001***	0.29 (0.16, 0.41), < 0.001***	0.22 (0.10, 0.35), < 0.001***
**Place of residence**				
In Shelter	1.00	1.00	1.00	1.00
With a family member/own home	0.90(0.71, 1.14),0.388	0.97(0.77, 1.23),0.811	−1.21(−3.92, 1.50),0.383	−0.41(−3.01, 2.28),0.768
With a friend/workplace	0.99(0.77, 1.26),0.925	1.02(0.79, 1.30),0.872	−0.27(−3.12, 2.58), 0.122	0.07(−2.74, 2.89),0.959
**Education Level**				
No education	1.00	1.00	1.00	1.00
Primary education	0,97(0.86, 1.08),0.551	0.95(0.85, 1.06),0.367	−0.36(−1.52, 0.79),0.539	−0.48(−1.61, 0.65),0.401
Sec education and above	1.09 (0.96, 1.24), < 0.001***	1.08(0.96, 1.22),0.201	0.89(−0.44, 2.22),0.192	0.78(−0.50, 2.07),0.231

*Others = Anglicans; *p < 0.05; **p < 0.01; ***p < 0.001.WW

### ACEs, trafficking vulnerability, and Post-traumatic stress disorder symptoms

Table 5 presents models estimating the associations between ACEs, trafficking vulnerabilities, and PTSD symptoms. In Model 1, each ACE was associated with a 6% increased risk of PTSD symptoms (aRR = 1.06; 95% CI: 1.03–1.09; p < 0.001). However, in Model 2, trafficking vulnerability was associated with a 9% reduced risk of PTSD symptoms (aRR = 0.91; 95% CI: 0.88–0.93; p < 0.001). Model 3 indicated a significant positive association between ACEs and PTSD symptoms (b = 1.80 per unit, 95% CI: 1.03–2.57, p < 0.001). Conversely, Model 4 showed that trafficking vulnerabilities were negatively associated with PTSD symptoms, with each additional unit of vulnerability leading to a decrease of 2.86 units in PTSD symptom severity (b = −2.86, 95% CI: −3.50 to −2.22, p < 0.001) (Table 5).

**Table 5:** Correlates of moderate to severe PTSD Symptoms (N = 350).

Variable	Adjusted relative risk ratio (95% confidence interval (CI), p-value		Unstandardized regression coefficient (95% CI), p-value	
	Model 1	Model 2	Model 3	Model 4
ACEs per unit	1.06(1.03, 1.09), < 0.001***		1.80(1.03, 2.57), < 0.001**	
Trafficking Vulnerabilities/Unit		0.91(0.88, 0.93), < 0.001***		−2.86(−3.50, −2.22), < 0.001***
**Marital Status**				
Single	1.00	1.00	1.00	1.00
Married	0.91(0.79, 1.05),0.192	0.95(0.83, 1.08),0.411	−2.76(−6.69, 1.16),0.168	−1.74(−5.46, 1.99),0.361
Widowed	0.88(0.69, 1.12),0.287	0.86(0.69, 1.07),0.193	−4.14(−11.12, 2.84),0.245	−4.41(−10.64, 1.81),0.164
**Religion**				
Christians	1.00	1.00	1.00	1.00
Moslems	1.01(0.89, 1.15),0.883	1.05(0.92, 1.18),0.473	0.34(−3.65, 4.34),0.866	1.22(−2.54, 4.97),0.526
Pent/ Saved	0.91(0.79, 1.04),0.173	0.95(0.83,1.07),0.391	−2.50(−6.23, 1.23),0.188	−1.63(−5.28, 2.02), 0.382
Others	1.09(0.96, 1.25),0.191	1.07(0.94, 1.22),0.276	2.79(−1.49, 7.09),0.202	2.05(−1.99, 6.09),0.563
**Age**, Per year	1.00(0.99, 1.01),0.924	0.99(0.98, 1.00),0.168	0.03(−0.35, 0.41),0.862	−0.26(−0.64, 0.12),0.182
**Place of residence**				
In Shelter	1.00	1.00	1.00	1.00
With a family member/own home	0.86(0.69, 1.07),0.172	0.95(0.75, 1.20),0.680	−5.58(−13.30, 2.15),0.157	−1.95(−10.76, 6.85),0.664
With a friend/workplace	0.89(0.71(1.14),0.379	0.93(0.72, 1.19),0.567	−4,32(−12.68, 4.05),0.312	−2.76(−12.08, 6.57),0.563
**Education Level**				
No education	1.00	1.00	1.00	1.00
Primary education	1.05(0.93, 1.18),0.419	1.02(0.92, 1.15),0.672	1.52(−2.06, 5.09),0.405	0.97(−2.43, 4.37),0.576
Sec education and above	1.03(0.89, 1.19),0.656	1.01(0.88, 1.16),0.839	1.03(−3.06, 5.20),0.630	0.57(−3.40, 4.55),0.778

*Others = Anglicans; *p < 0.05; **p < 0.01; ***p < 0.001.

## Discussion

This study examined the relationship between Adverse Childhood Experiences (ACEs), re-trafficking vulnerability, and mental health outcomes (anxiety, depression, and PTSD) among female survivors of human trafficking in Kampala, Central Uganda. The findings from this study indicated significant associations between anxiety, depression, and PTSD, while the risk of re-trafficking (re-trafficking vulnerability) was negatively associated with mental health symptoms.

The study revealed high rates of mental health problems among the survivors, with over 57.1% of the survivors reporting anxiety symptoms, 56.0% reporting depression symptoms, and 40.9% reporting PTSD symptoms, consistent with prior studies on the impact of trafficking and other traumatic experiences on the survivors [8,40]. Earlier studies in low-resource settings, particularly in regions with limited access to mental health services, have highlighted the burden of mental health challenges faced by survivors of human trafficking [8,40]. In Uganda, where mental health services are scarce with social stigma surrounding mental health [41,42], the reported rates of mental health symptoms are of high concern. Besides, the limited access to trauma-informed care increases the psychological distress survivors face, preventing many from receiving the necessary support and treatment they need for recovery. More so, the high prevalence of mental health problems reveals the need for the integration of culturally sensitive trauma-informed mental health services within the shelters and reintegration programs for survivors of trafficking.

The lack of accessible trauma-informed care exacerbates the psychological burdens these women face, preventing many from receiving the necessary treatment and support for recovery. Moreover, the high prevalence of mental health conditions indicates an urgent need for integrating mental health services into the care systems for trafficking survivors, particularly within shelters and reintegration programs.

The study’s results indicate a clear link between ACEs and heightened vulnerability to mental health disorders. Specifically, the adjusted risk ratios (aRR) for anxiety (aRR = 1.05, P < 0.001), depression (aRR = 1.04, P = 0.002), and PTSD (aRR = 1.06, P < 0.001) were significantly higher among those with a history of ACEs. These findings support the robust body of literature that links childhood adversity to long-term psychological distress and greater susceptibility to exploitation, including human trafficking [11,43].

In Uganda, where family separation, domestic violence, and orphanhood due to HIV/AIDS are widespread [44], ACEs are common and often go unaddressed. These early life stressors leave individuals more vulnerable to further exploitation later in life, as they may lack the coping skills, emotional resilience, and social support necessary to protect themselves from trafficking. The study highlights the relevance of early interventions tailored towards preventing ACEs and mitigating their lasting impacts. Comprehensive child protection strategies and family support programs could reduce the risk of trafficking and its associated mental health consequences.

One of the key findings of this study was the high level of trafficking vulnerability among participants, with 63.4% of survivors showing high levels of vulnerability to re-trafficking. This is consistent with the broader literature that identifies younger women, particularly those aged 18–24 years, as being at higher risk for trafficking, including those who have been formerly trafficked due to factors such as economic dependence, lack of social support, and limited education [7,8]. These vulnerabilities are compounded by the intersection of poverty, restricted access to education, and the challenges of reintegration into society after trafficking experiences.

The study found a paradoxical negative relationship between trafficking vulnerability and mental health outcomes, with higher vulnerability associated with reduced risk of PTSD, depression, and anxiety. Several factors may explain this unexpected finding: survivors may develop psychological resilience and coping mechanisms, such as emotional numbing or detachment, that mitigate the expression of mental health symptoms without indicating overall well-being [45]; cultural stigma surrounding mental health in Uganda may lead to underreporting of distress, as individuals suppress symptoms to avoid discrimination; and for some survivors, particularly those trafficked multiple times, there may be a normalization of exploitation, which reduces the perceived psychological impact of trafficking. These factors underscore the complexity of mental health in trafficking survivors and highlight the need for a nuanced understanding of how resilience, coping, and cultural contexts influence psychological responses to trauma.

While the Adult Human Trafficking Screening Tool (AHTST) is widely used across health and social service settings, formal psychometric validation remains limited. Federal developer materials explicitly note that it has not yet been validated, though it was built from best practices and related instruments. However, recent work has shown promising results in evaluating modified AHTST forms in displaced/refugee populations, demonstrating a high test accuracy. The broader adult screening literature (e.g., the validated TVIT) supports the underlying constructs of coercion and exploitation captured by our items.

In our context, higher AHTST scores co-occurred with lower symptom severity. One possibility is a measurement-process explanation: survivors engaged in services may develop greater trafficking awareness and thus endorse situational-risk items more readily while concurrently under-reporting internalizing symptoms due to stigma, avoidance/numbing, or response shifts. A second, not mutually exclusive, possibility is resilient adaptation/post-traumatic growth, whereby some survivors report fewer current symptoms despite ongoing vulnerability. Future work should locally validate the AHTST in Uganda, including its factor structure, reliability, and cultural/linguistic equivalence, and integrate qualitative cognitive interviewing to optimize cultural applicability.

Counter to some high-income settings where education is protective for depression, our data indicated higher depressive symptom risk with increasing age and among participants with ≥secondary education. In Low and Middle-Income Countries (LMIC) contexts, education can coexist with unemployment, income precarity, and unmet expectations, all linked to common mental disorders; more educated survivors may also recognize and report symptoms more readily due to greater mental-health literacy. In trafficking survivors specifically, older age may reflect cumulative trauma exposure and increased reintegration burdens. Together, these pathways could explain the observed pattern. Future analyses should examine age-education interactions and longitudinal changes as survivors stabilize socioeconomically.

### Study limitations

Much as this study highlights Adverse childhood experiences (ACEs), re-trafficking vulnerability, and mental health outcomes, it has several limitations that are worth noting. The study adopted a cross-sectional design, limiting the potential to recognize the cause-and-effect relationship between ACEs, mental health outcomes, and re-trafficking vulnerability. Future longitudinal research is needed to track mental health and vulnerability changes over time.

Further, the current study relied on self-reported data, subject to recall and social desirability biases. Respondents may have underreported or over reported their experiences due to stigma or difficulty recalling past events accurately. Third, the study was conducted among survivors receiving services from shelters and organizations, which may not be representative of all trafficking survivors in Uganda, particularly those who have not accessed support services. This selection bias may have influenced the findings. Lastly, this study did not profoundly explore cultural factors affecting mental health reporting and coping mechanisms. Qualitative research could complement these findings by providing deeper insights into how survivors perceive and navigate their mental health challenges in different socio-cultural contexts. We relied on validated self-report screeners (PHQ-9, GAD-7, PCL-5) to estimate symptom burden; these instruments are not equivalent to clinician-administered diagnostic interviews. As such, prevalence and associations may differ from estimates based on structured clinical diagnostics, particularly in settings with variable mental-health literacy and stigma. We made significant efforts to reduce misclassification by modeling symptom severity as continuous outcomes alongside standard cutoffs; nonetheless, residual measurement error remains possible.

### Implications for policy, practice, and research

The results of this suggest several critical policy and practice interventions to address the vulnerabilities of women and girls to trafficking and their mental health needs. Expanding access to trauma-informed mental health services, particularly within shelters and community-based programs, is essential, alongside the integration of peer support networks to enhance emotional well-being. Prevention of Adverse Childhood Experiences (ACEs) through improved child protection, parenting support, and community engagement is vital to mitigate future trafficking risks. Socioeconomic empowerment programs, including vocational training, livelihood opportunities, and financial literacy, can reduce economic pressures that make individuals vulnerable to exploitation. Strengthening legal frameworks by ensuring victim-centered legal support, enhancing inter-agency coordination, and reinforcing the enforcement of anti-trafficking laws are key steps in both protecting survivors and holding traffickers accountable. A holistic, integrated approach that combines mental health care, prevention, empowerment, and legal support is necessary to mitigate the lasting effects of trafficking and support the recovery of survivors.

This study highlights several key areas for future research to enhance the understanding and support of trafficking survivors. Longitudinal studies tracking the mental health trajectories of survivors are needed to identify the evolving nature of mental health conditions post-trafficking, providing insights into long-term recovery and the effectiveness of interventions. Additionally, investigating coping mechanisms and resilience factors could offer valuable information on how survivors navigate the psychological impact of adverse childhood experiences (ACEs) and trafficking, potentially informing strategies for fostering psychological recovery. Finally, research into community-based interventions is essential to developing culturally appropriate and sustainable models for preventing re-trafficking and supporting reintegration, focusing on understanding how community dynamics influence recovery and long-term well-being.

## Conclusions

This study provides critical insights into the complex link between ACEs, trafficking vulnerability, and mental health among female survivors in Uganda. The findings underscore the urgent need for a multi-faceted approach to addressing human trafficking, one that includes trauma-informed care, prevention of childhood adversity, socio-economic empowerment, and robust legal protections. By addressing the root causes of trafficking and improving the mental health outcomes of survivors, Uganda can take significant strides toward breaking the cycle of exploitation and promoting the well-being of trafficking survivors.

## Supporting information

S1 Dataset This is the dataset containing anonymized participant-level data used in the analyses presented in this study. (CSV)

S2 File Renamed be7eb. This is the PLOS’ Questionnaire on inclusivity in global research. (DOCX)
